# Using tramadol to monitor hepatic drug metabolism in the critically ill

**DOI:** 10.1186/cc10950

**Published:** 2012-03-20

**Authors:** K Lane, JJ Dixon, D McKeown, A Johnston, I MacPhee, BJ Philips

**Affiliations:** 1 Acute Kidney Injury Research Group, St George's, University of London, UK; 2 Analytical Services International Ltd, St George's, University of London, UK

## Introduction

Previously, we have demonstrated significant inhibition of hepatic drug metabolism by the enzymes cytochrome P450 (CYP) 3A4 and 3A5 in acute kidney injury (AKI) using midazolam as a probe drug [1,2]. We are now developing the use of tramadol as a probe drug to test the hypothesis that CYP2D6 function is also inhibited by AKI in critical illness. In this study we sought to determine whether a single timepoint tramadol concentration could be identified as a reliable surrogate for measurement of a full area under the concentration time curve after intravenous administration in adults.

## Methods

We conducted a study of 10 critically ill patients in our hospital's general critical care unit. Tramadol 10 mg was given intravenously, and serum was taken at 0.5, 1, 2, 3, 4 and 8 hours for determination of concentrations of tramadol ([tramadol]) and its two main metabolites. Inclusion criteria: age >18 years, predicted ICU stay >48 hours. Exclusion criteria: recent receipt of tramadol or major CYP2D6 inhibitors, hepatic failure, pregnancy/breastfeeding.

## Results

**Figure 1:**
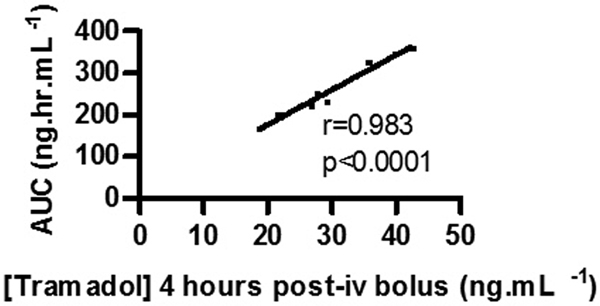
**Correlation of [tramadol] at *t *= 4 hours and AUC [tramadol]-time graph. iv, intravenous**.

There was a strong correlation between the area under the curve (AUC) of the [tramadol]-time graph and *t *= 4 hours [tramadol], *P *< 0.0001, *r *= 0.983. See Figure 1. The [tramadol] at other timepoints correlated less strongly with the AUC. The mean [tramadol] at 4 hours was 29.7 ng/ml (24.3 to 35.1) and the mean AUC was 257 ng/hour/ml (211 to 303). Analysis of tramadol metabolites confirmed that CYP2D6 was predominantly responsible for tramadol metabolism.

## Conclusion

A single blood sample, taken 4 hours post-intravenous tramadol injection, reliably predicts integral tramadol exposure in critically ill adults and may be useful for assessing CYP2D6 function.

A larger study of the influences of AKI and CYP genotype on hepatic drug metabolism in the critically ill is underway.
